# A Predictive Noninvasive Single-Nucleotide Variation–Based Biomarker Signature for Resectable Pancreatic Cancer: Protocol for a Prospective Validation Study

**DOI:** 10.2196/54042

**Published:** 2024-05-13

**Authors:** Nico Seeger, Stefan Gutknecht, Irin Zschokke, Isabella Fleischmann, Nadja Roth, Jürg Metzger, Markus Weber, Stefan Breitenstein, Lukasz Filip Grochola

**Affiliations:** 1 Department of Visceral and Thoracic Surgery Cantonal Hospital of Winterthur Winterthur Switzerland; 2 Department of Visceral, Thoracic and Cardiovascular Surgery Triemli Hospital Zurich Switzerland; 3 Department of General and Visceral Surgery Cantonal Hospital of Lucerne Lucerne Switzerland

**Keywords:** single-nucleotide polymorphism, SNP, single-nucleotide variation, SNV, pancreatic ductal adenocarcinoma, PDAC, noninvasive biomarker, survival, resection, prospective validation

## Abstract

**Background:**

Single-nucleotide variations (SNVs; formerly SNPs) are inherited genetic variants that can be easily determined in routine clinical practice using a simple blood or saliva test. SNVs have potential to serve as noninvasive biomarkers for predicting cancer-specific patient outcomes after resection of pancreatic ductal adenocarcinoma (PDAC). Two recent analyses led to the identification and validation of three SNVs in the CD44 and CHI3L2 genes (rs187115, rs353630, and rs684559), which can be used as predictive biomarkers to help select patients most likely to benefit from pancreatic resection. These variants were associated with an over 2-fold increased risk for tumor-related death in three independent PDAC study cohorts from Europe and the United States, including The Cancer Genome Atlas cohorts (reaching a *P* value of 1×10^–8^). However, these analyses were limited by the inherent biases of a retrospective study design, such as selection and publication biases, thereby limiting the clinical use of these promising biomarkers in guiding PDAC therapy.

**Objective:**

To overcome the limitations of previous retrospectively designed studies and translate the findings into clinical practice, we aim to validate the association of the identified SNVs with survival in a controlled setting using a prospective cohort of patients with PDAC following pancreatic resection.

**Methods:**

All patients with PDAC who will undergo pancreatic resection at three participating hospitals in Switzerland and fulfill the inclusion criteria will be included in the study consecutively. The SNV genotypes will be determined using standard genotyping techniques from patient blood samples. For each genotyped locus, log-rank and Cox multivariate regression tests will be performed, accounting for the relevant covariates American Joint Committee on Cancer stage and resection status. Clinical follow-up data will be collected for at least 3 years. Sample size calculation resulted in a required sample of 150 patients to sufficiently power the analysis.

**Results:**

The follow-up data collection started in August 2019 and the estimated end of data collection will be in May 2027. The study is still recruiting participants and 142 patients have been recruited as of November 2023. The DNA extraction and genotyping of the SNVs will be performed after inclusion of the last patient. Since no SNV genotypes have been determined, no data analysis has been performed to date. The results are expected to be published in 2027.

**Conclusions:**

This is the first prospective study of the *CD44* and *CHI3L2* SNV–based biomarker signature in PDAC. A prospective validation of this signature would enable its clinical use as a noninvasive predictive biomarker of survival after pancreatic resection that is readily available at the time of diagnosis and can assist in guiding PDAC therapy. The results of this study may help to individualize treatment decisions and potentially improve patient outcomes.

**International Registered Report Identifier (IRRID):**

DERR1-10.2196/54042

## Introduction

Pancreatic ductal adenocarcinoma (PDAC) is the most frequent type of pancreatic cancer and one of the most lethal malignancies due to a tendency for early local invasion and high metastatic potential [1,2]. Despite this aggressive phenotype, PDAC shows a wide range of biological features, whereby early recurrence is observed in some patients who undergo pancreatic resection, whereas long-term cancer-free survival can be achieved in others [3-5]. It is considered that such individual oncologic courses and the response to personalized treatment plans can be predicted by gaining a more comprehensive understanding of human germline genetic variation [6,7]. Importantly, this genetic information can be found in the germline DNA and thus does not require a tumor biopsy or the technically demanding isolation of circulating tumor cells. Instead, samples can be easily obtained by a simple and cheap blood or saliva test at any time during diagnostic workup or treatment, offering a noninvasive predictive biomarker available at the time of diagnosis. Indeed, this type of personalized medicine is no longer a fantasy but a maturing reality. In other cancer types, including acute lymphoblastic leukemia, colorectal cancer, lung cancer, and breast cancer, the genotyping of certain single-nucleotide variations (SNVs; formerly SNPs) is recommended by the US Food and Drug Administration [8].

A growing body of evidence suggests that inherited genetic variants can also be used to predict cancer-specific patient outcomes and guide therapy for patients with pancreatic cancer. Specifically, in a retrospective analysis, we identified a germline variant in the CD44 gene (rs187115) that can serve as a predictive biomarker to help select patients who are likely to benefit from pancreatic resection [9,10]. More recently, we performed a genome-wide screen for inherited variants that affect survival in patients with PDAC based on existing biological knowledge about the regions where the genetic variants reside [11]. This search for high-frequency polymorphic variants that affect tumor-related survival and either result in an altered protein structure and function or reside in known regulatory noncoding genomic regions has identified two clinically relevant sequence variants in functional regions of genes that are known to regulate cancer progression, invasion, and metastasis [11]. Specifically, regulatory variants within *CHI3L2* (rs684559) and *CD44* (rs353630) were found to be associated with an over 2-fold increased risk of tumor-related death in two independent retrospective PDAC study cohorts from Europe and the United States (hazard ratio [HR] 0.38, 95% CI 0.27-0.53; *P*=1×10^–8^, q value<.05) [11].

Taken together, these retrospective studies have demonstrated the potential of polymorphic sequence variants to identify a subset of patients with high-risk pancreatic cancer associated with a very low survival probability that might be eligible for inclusion in clinical trials of new therapeutic strategies, including neoadjuvant chemotherapy protocols and novel adjuvant systemic regimens. In addition, the findings from these studies suggest that the biological knowledge about these SNVs could help guide the development of such individualized genomic treatment strategies.

However, despite their robustness, the described results have not yet been validated prospectively and it is unclear whether these observations can be independently replicated in a controlled clinical setting.

## Methods

### Study Aim, Design, and Setting

**Figure 1:**
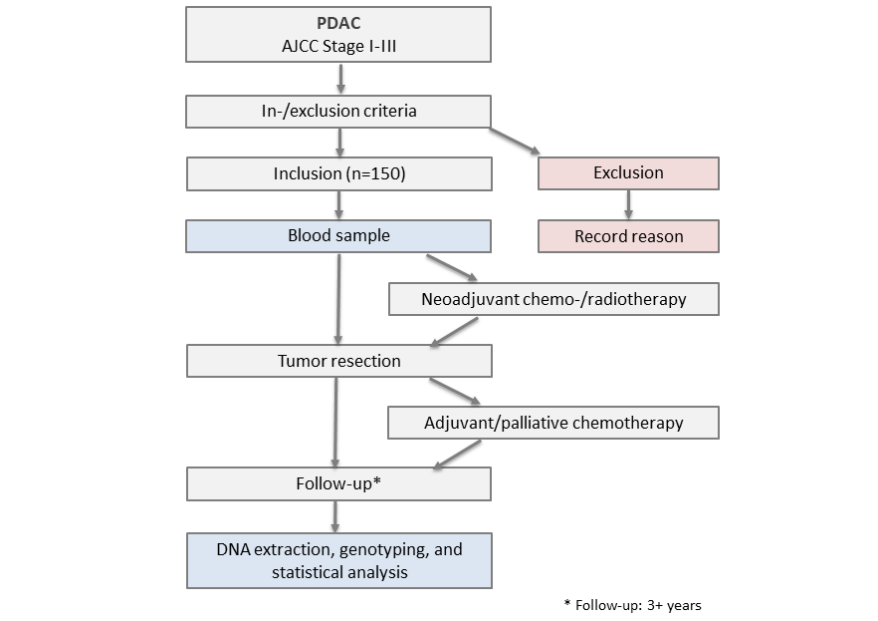
Study outline. A planned sample of 150 consecutive patients with histopathologically confirmed resectable pancreatic ductal adenocarcinoma (PDAC) will be treated at three designated Swiss hospitals according to the best standard of care and prospectively included in the study. The first patient was included in August 2019 and the recruitment is ongoing. The estimated end of data collection is May 2027. The survival analysis using the single-nucleotide variation (SNV) genotyping data after DNA extraction from the blood collected at the time of inclusion will be performed after completion of the follow-up of all patients to ensure that all study participants and physicians are fully blinded with regard to the SNV genotypes of the patients. AJCC: American Joint Committee on Cancer.

This project is designed as a one-arm prospective controlled clinical study that aims to validate the association of the previously identified SNVs in the CD44 and CHI3L2 genes with PDAC survival after tumor resection. We aim to use a prospective cohort of patients with PDAC who will undergo pancreatic resection at our established network of three cantonal hospitals (Departments of Surgery at the Cantonal Hospital of Winterthur, Cantonal Hospital of Lucerne, and the Triemli Hospital in Zurich) in Switzerland. The study outline is depicted in Figure 1.

### Participant Characteristics

Patients with a diagnosis of a pancreatic tumor mass that is either biopsy-proven or highly indicative of PDAC will be recruited before surgery in the outpatient clinics of the participating centers after completed standard preoperative evaluation of a pancreatic cancer diagnosis and assessment of tumor resectability according to the best standard of care and the following criteria: (1) pancreatic tumor mass either biopsy-proven or highly indicative (as assessed by preoperative magnetic resonance imaging, computed tomography, and/or endoscopic ultrasound imaging) of PDAC in the head or tail of the pancreas; (2) tumor, node, and metastasis stage c/uT1-4, c/uNx, cM0 (according to the 7th edition of the American Joint Committee on Cancer staging system); (3) open or minimally invasive pancreatic resection, including conversion laparotomy; (4) underwent primary tumor resection; (5) received neoadjuvant chemotherapy and/or radiochemotherapy; (6) previously underwent pancreatic resection for reasons other than PDAC; (7) age≥18 years; and (8) provided written informed consent. Patients who subsequently successfully undergo tumor resection and whose diagnosis of PDAC is confirmed by histopathological examination of the resected specimen will be included in the study. All patients who fulfill these criteria will be included, regardless of the tumor resection margins (R0, R1, R2) and regardless of intra- or postoperative mortality.

The presence of one of the following criteria will lead to the exclusion of the patient: (1) any histopathological entity other than PDAC, including but not limited to adenocarcinoma of the papilla of Vater, acinar cell carcinoma, distal cholangiocarcinoma, and neuroendocrine tumors such as neuroendocrine carcinoma; (2) metastatic disease, including intraoperative findings confirmed by histopathological examination (cM1, pM1); (3) previous pancreatic resection for PDAC and presenting either with local recurrence or a second primary tumor in the pancreatic remnant, but not yet included in the study; (4) age<18 years; and (5) did not provide written informed consent. With respect to exclusion criterion (3), individuals who had already been included in the study at the time of the first resection of a PDAC will not be excluded from the study retrospectively because of a local recurrence or a second primary tumor but will continue to receive follow-up. All patients that will be excluded from participation will be recorded together with the reason for the exclusion.

### Endpoints and Data Collection

The primary endpoint of the study is tumor-related survival. Secondary endpoints include recurrence and overall survival. The exact type and timing for data and blood sample collection are detailed in Table 1. The estimated total duration of the study is 5 years, including the scheduled 3-year postoperative follow-up.

**Table 1:** Type and timing of data and blood sample collection.

Data and timing of collection	Screening	Surgery	Early postoperative period	Follow-up
Visit	1	2	3	4, 5, and 6
Time	Preoperative	day 0	1 week postoperative	1, 2, and 3 years
Participant information and informed consent	✓			
Medical history	✓			
Inclusion/exclusion criteria check	✓			
Blood collection	✓			
Surgical procedure		✓		
Histopathological data			✓	
Recurrence/survival data				✓

All cases will be presented at interdisciplinary tumor board meetings at each participating center pre- and postoperatively. All patients will be offered standard-of-care multimodal treatment at the discretion of the interdisciplinary tumor board at each participating center. The operation will be performed according to the group assignment by senior surgeons only, who have extensive experience in pancreatic resections. All surgeries will be performed at the Departments of Surgery at the Cantonal Hospital of Winterthur, Cantonal Hospital of Lucerne, and the Triemli Hospital in Zurich. The follow-up of the patient will be recorded for at least 3 years after tumor resection. Data collection will be completed using the hospital records or by contacting the patient’s general practitioner or oncologist; thus, no additional appointments in the outpatient clinic will be required for study purposes.

### Power of the Study

Sample size calculations were performed in G-Power 3.1 software (Heinrich-Heine University, Germany) based on the results of the previously mentioned retrospective studies according to the ability to detect an HR>2 between genotypes [9-11]. With the use of a two-sided α level of .05 and considering a dropout rate of 10% as well as a potential additional error margin of 10%, 150 patients are required to obtain 80% power to detect a difference in cancer-related survival between genotypes.

### Statistical Analysis

A parametric two-sided Student *t* test and a two-tailed Mann-Whitney *U* test will be used to compare data distributions in groups with normally distributed data and data that deviate from a normal distribution, respectively. The Fisher exact test will be used to calculate frequency distributions in cross-table analyses. Kaplan-Meier survival curves (log-rank test) and Cox proportional hazards models will be calculated to assess and compare survival times. All statistical analyses will be performed using R and SPSS software.

### Ethical Considerations

The study will be carried out in accordance with principles enunciated in the current version of the Declaration of Helsinki, the guidelines of Good Clinical Practice (GCP) issued by the International Council for Harmonisation of Technical Requirements for Pharmaceuticals for Human Use, and the requirements of the Swiss regulatory authority. The study was approved by the local ethics committee (Kantonale Ethikkommission Zürich, BASEC-Nr. 2019-00178) on April 18, 2019. All patients will be asked to sign a written informed consent form that has been approved by the ethics committee, which describes in detail the aims and methods of the study as well as lists all potential risks posed to the participants. Privacy and confidentiality are fully ensured since the study data are anonymous and deidentified. All data relevant to this study will be coded (including biological material and health-related personal data), safely protected, and recorded in a digital format using a GCP-compliant electronic data capture system. The study participants will receive no financial compensation for participating in the study.

## Results

The follow-up of the patients started in August 2019. The estimated end of data collection is May 2027. Recruitment is ongoing. As of November 2023, 142 patients have been recruited.

The DNA extraction and genotyping of the SNVs will be performed after the inclusion of the last patient. The data analysis will be performed at the end of follow-up of the last patient. Since no SNV genotypes have been determined, no data analysis has been possible to date. The results are expected to be published in 2027.

## Discussion

This is the first prospective study of the clinical value of *CD44* and *CHI3L2* SNV–based biomarkers in PDAC after tumor resection. The design of this study enables minimizing the biases inherent in retrospective trials, such as selection or publication biases. Moreover, akin to a randomized controlled trial designed to compare clinical interventions, both the study participants and all physicians involved in the treatment of the included patients will be fully blinded by determining the SNV genotypes and analyzing the results only after the completion of follow-up of all patients. Such a prospective validation of these biomarkers would allow for their clinical use as a predictive signature of survival after pancreatic resection that can be readily available at the time of PDAC diagnosis.

The main limitation of the study is the heterogeneity of the patient group with regard to the neoadjuvant and adjuvant treatment(s) the individual participants will receive. These treatments are known to affect patient outcomes, including cancer-related survival as the main endpoint of this study. However, this heterogeneity also reflects real-world clinical practice and should thus provide a robust biomarker that can be applied to all patients with PDAC who undergo resection of their tumors, regardless of the chemoradiotherapy they individually receive. Another limitation is the disruption of administrative and processes during the COVID-19 pandemic. However, since patients diagnosed with pancreatic cancer received adequate treatment without major delays and were included consecutively in this study during this disruptive period, the effect on the study results should be negligible.

The genotype of the identified SNVs can be determined before the start of any treatment by a technically simple blood or even saliva test, which can then be used to identify patients who are at higher risk for faster tumor progression [6,9]. Other prognostic factors currently in use or suggested to be useful in a clinical setting such as completeness of resection, lymph node involvement, or postoperative carbohydrate antigen 19-9 serum levels can only be precisely determined after surgical resection and therefore have limited relevance to treatment decisions [12]. In contrast, SNV-based biomarkers can guide preoperative treatment decisions such as the decision to perform neoadjuvant chemotherapy in patients with an aggressive tumor biology or can affect adjuvant treatment protocols. The results of this prospective study may lead to improved patient outcomes in patients with PDAC that is amenable to resection.

## Abbreviations

GCP: Good Clinical Practice
HR: hazard ratio
PDAC: pancreatic ductal adenocarcinoma
SNV: single-nucleotide variation
